# The American International Congress on Tuberculosis

**Published:** 1906-05

**Authors:** 


### TEXAS MEDICAL JOURNAL.

AUSTIN, TEXAS.

A MONTHLY JOURNAL OF MEDICINE AND SURGERY.

EDITED AND PUBLISHED BY
F. E. DANIEL, M. D.
Mrs. F. E. DANIEL, Business Manager.

Published Monthly at Austin, Texas. Subscription price $1.00 a year in advance

### THE AMERICAN INTERNATIONAL CONGRESS ON TUBERCULOSIS.

The fourth biennial sitting of the American International Congress on Tuberculosis will take place in New York City, November 14, 15, and 16, 1906, under the auspices of the Medico-Legal Society.

The American Congress on Tuberculosis was organized in February, 1900, and held its first session in New York in June, 1900; its second in New York in June, 1902; its third in St. Louis in October, 1904, at which time, owing to the international character given to it by representatives from foreign countries in response to invitations sent by Mr. Hay, Secretary of State, through our foreign diplomats, the name of the Congress was changed to "The American International Congress on Tuberculosis."

At the 1902 meeting in New York, in consequence of some indiscretion on the part of the management and a misconception, certain parties, objectionable to the regular profession, were permitted to participate in the deliberations, and in consequence thereof there was a split off, much to be regretted, and a separate Congress was organized by the seceders, under the presidency of Dr. Lewis, who later resigned. Dr. Brown, of Atlanta, Ga., was elected president, and the new Congress, under his management, held a large and interesting meeting at Atlanta, Ga., in April, 1904. It was to have met at El Paso, Texas, in 1905, but no meeting was held, and the organization, so far as I know, has been abandoned.

The parent Congress, the original organization, now the American International, was the first in the field in the great humanitarian work so pressing and so vital, and set in motion the vigorous crusade against the great foe to mankind, and has done great and lasting work. At the St. Louis meeting in October, 1904, there was a large and able and enthusiastic attendance, and the fraternities, in their national organization numbering some five and a half million members, and the Association of Engineers, and other bodies interested in the public health and especially the control of consumption, joined forces with the Congress, and a campaign was mapped out and will be vigorously prosecuted at the coming sitting in New York in November. The work will be mainly in the direction of securing legislation to enforce sanitary measures for the prevention of the spread, and as far as may be, the removal of the causes of the disease,— unsanitary environments, life and occupation of the masses. It is unnecessary to here go into an account of the decline in cases, and diminution in the deaths in New York City alone, in consequence of improved sanitation in the dwellings and factories and work shops,—the lives and environments of the toilers, the passing of the sweat-shops, the dark, damp and overcrowded tenement houses, the opening up of wide streets, the letting in of sun and air, etc.

The prevention of the communication of the disease from one person to another would seem to be easily possible, as it is in no sense contagious, by the destruction of the sputum and other secretions which contain the infective principle, but the eradication of the disease is Utopian, it being a disease of our civilization—a house disease. But the causes that produce it can largely be removed. Better food, better living, the observance of the rules of health, air, light, and pure water, will accomplish much in this direction. And the people must be enlightened.

Already a large attendance upon this Congress is assured; and many papers by well-known authorities have been secured. There will be a large foreign representation and much good is anticipated to result from the conference.

The policy of the management is, to conciliate and reconcile all persons, lay and professional, who are interested and engaged in the crusade against consumption. All are invited to participate and become members of the Congress — physicians, sanitarians, educators, editors, engineers, statesmen, architects — all whose labors and influence can contribute to the accomplishment of the great end in view. The Congress recognizes no faction or rival, antagonizes no movement on the part of others, no organization engaged in this work, but desires to unify the work by co-operating with and securing the co-operation of all such organizations. It rejoices in the success of the great Anti-Tuberculosis League, and will gladly lend its aid, while inviting its co-operation. There is, and should be, no rivalry. Let every lover of his kind contribute what he may to the advancement of this great humanitarian work —the throttling of the monster responsible for the greatest mortality amongst mankind.

